# Technology-Enabled Active Learning: Assessment of Dentistry Students’ Perception of Digital Prosthodontic Workflow

**DOI:** 10.3390/dj13040138

**Published:** 2025-03-24

**Authors:** Navodika Yaparathna, Iresha Udayamalee, Megan Gray, Andrew Cameron, Jane Evans, Menaka Abuzar

**Affiliations:** School of Medicine and Dentistry, Griffith University, Gold Coast Campus, Southport, QLD 4215, Australia; i.udayamalee@griffith.edu.au (I.U.); megan.gray@griffith.edu.au (M.G.); a.cameron@griffith.edu.au (A.C.); j.evans@griffith.edu.au (J.E.)

**Keywords:** active learning, assessment tool, CAD/CAM, digital curriculum, dental education, feedback

## Abstract

**Background/Objectives**: Adoption of digital dentistry into curricula by higher education institutions has become mandatory. Implementing changes in stringent and crowded curricula requires meaningful evaluation. The current study aimed to assess the psychometric properties of an adapted Technology-Enabled Active Learning (TEAL) questionnaire on students’ perception of integrating a digital workflow in the undergraduate curriculum, and to evaluate students’ perception of benefits of computer-aided design/computer-aided manufacturing (CAD/CAM) in dentistry. **Methods**: Dental students engaged in hands-on experience in digital fabrication of a dental crown. The study was conducted in two stages in consecutive years as follows. Stage 1: Validation of the adapted TEAL questionnaire in an Australian dental school with 110 undergraduate (UG) students. Content and construct validity were assessed using mixed methods. Reliability was ensured with Cronbach’s Alpha. Stage 2: Qualitative and quantitative analysis of students’ perception on implementation of digital workflow with 140 students using the adapted TEAL questionnaire. **Results**: The Content Validity Index for the adapted TEAL questionnaire was 0.74, and qualitative analysis displayed positive sentiment. Structural equation modelling showed absolute, relative, and Parsimony fit indices of RMSEA value of 0.055, SRMR of 0.070, GFI of 0.837, and CFI of 0.979. Cronbach’s Alpha was 0.952. Most students (93.75%) had a positive attitude towards digital workflow. The qualitative analysis revealed implementing digital workflow in the dental curriculum has a positive impact on developing digital skills. **Conclusions**: The adapted TEAL questionnaire construct has good psychometric properties in dentistry students’ context. It can be utilised as a valid and reliable method to ascertain students’ active learning experience of digital workflow. The majority of the students had positive perceptions on the integration of digital workflow.

## 1. Introduction

Digital literacy and technologies are fast replacing traditional analogue techniques, ushering in a new era in dentistry. This seamless integration of digital workflow in patient-centred care offers multiple benefits, such as increased efficiency and improved treatment outcomes in an operator- and patient-friendly manner [1]. Among digital workflows, CAD/CAM is paramount for manufacturing indirect restorations such as dental appliances, fixed prostheses, and removable prostheses [2,3]. Digital workflows are now considered a standard clinical protocol in modern clinical dental practice.

Simultaneously, the undergraduate (UG) learning paradigm has evolved to embrace technology-enhanced active learning strategies to improve the student’s learning experience [4]. It can therefore be envisioned that the application of technologies in digital workflow could be merged with dynamic learning strategies in the undergraduate dental curriculum. Embedding digital technology into the contemporary dental curriculum has facilitated learning opportunities not previously available to students, aiding in their understanding of techniques and work readiness [5]. As a result, the implementation of digital technologies in undergraduate dental education should be viewed as a critical component of a dental students’ education [6,7].

Undergraduate (UG) dental curricula need to be revised regularly to prepare students to fulfil the demands of the current profession and improve the employability outcomes of dental graduates domestically and internationally [8]. Embracing a digital perspective across dental education will foster students’ learning to graduate confidently and eventually become more competent in digital dentistry, facilitating them to succeed in a competitive job market [5]. Moreover, learning CAD/CAM technology throughout the UG curriculum in an authentic setting will likely strengthen collaboration opportunities between students studying dentistry, dental prosthetics and dental technology [9]. This interdisciplinary approach will garner innovation and exchange of each other’s expertise. However, as this is a novel area, assessing how the implementation will affect UG students’ knowledge, learning outcomes, and satisfaction with the preclinical prosthodontic curriculum is vital. The current literature evidenced a lack of comprehensive research to assess how the students in this digital era embrace learning CAD/CAM dentistry.

There are limited valid and reliable instruments to assess the undergraduates’ Technology-Enabled Active Learning (TEAL) construct. The TEAL scale was initially developed and validated by Shroff et al. in 2019 with first-year undergraduate students at the Polytechnic University, Hong Kong, and it exhibited good psychometric properties in a nondental context [10]. The scale consists of 20 latent variables organised into 4 latent factors: interactive engagement, problem-solving skills, interest, and feedback [10]. After scrutiny, the team selected the TEAL scale to assess the construct of UG students’ perception of digital dental education in an Australian context.

The objectives of this study were as follows: to adapt the TEAL questionnaire to the dental learning context and validate it, assess students’ perceptions of integrating the digital workflow into the preclinical prosthodontics dentistry curriculum, and to evaluate students’ perception of benefits of computer-aided design/computer-aided manufacturing (CAD/CAM) in dental practice.

## 2. Materials and Methods

The study participants were all UG dentistry students enrolled in the third-year preclinical fixed prosthodontics course. The research was conducted over two consecutive years. During the first year, the TEAL questionnaire was adapted to meet the objectives of the study and validated (stage I). The validated questionnaire was then administered to the third-year cohort of the following year (stage II). Ethical approval for the study was granted by the Human Ethics Committee of the University (GU Ref No 2022/854 on 16/09/2022).

Written informed consent was obtained from all the participants before administering the questionnaire. Participation was voluntary, and no incentives were offered.

### 2.1. Learning Activity

A preclinical prosthodontics digital workflow was designed and implemented for sixteen weeks, with tasks underpinning knowledge for psychomotor learning spanning online lectures for theoretical content, short videos uploaded on students’ learning platform, and small group practical sessions for hands-on experience. All participants (two cohorts of students in consecutive years) completed molar crown preparations on typodont models in phantom heads in a simulated environment. Polyvinyl siloxane impressions were made using a custom tray, and models were fabricated. With supervision from qualified dental technicians, students scanned the models using a desktop scanner (E3, 3Shape B/V, Copenhagen, Denmark). The crown was designed using CAD software (Dental system 2022, 3Shape B/V, Copenhagen, Denmark) and milled (PrograMill PM7, Ivoclar, Schaan, Lichtenstein) from poly methyl methacrylate (PMMA). The students finished and polished their crowns following standard protocols. The crowns were checked for proximal contacts, occlusion, and margins on the original models and cemented with a resin-modified glass ionomer luting cement. The individual reflective feedback was provided to students during and after each step.

Additionally, students (in groups of 16) observed the manufacturing of crowns using a 3D printer (Asiga Max UV, Sydney) by qualified dental technicians to ensure exposure to a different technology.

### 2.2. Stage I: Adaptation and Validation of TEAL Questionnaire for Dentistry Learning

During stage I, a mixed method was used to adapt and validate the TEAL questionnaire to meet the objectives of the study. The face and content validity of the questionnaire was evaluated with quantitative and qualitative input obtained from academics and clinicians. The construct validity, reliability, and acceptability were evaluated using the students’ perceptions.

The TEAL scale’s content validity was assessed on its face value, clarity, construct, and relevance by a panel of professionals (*n* = 10) with expertise in contemporary methods in dentistry, undergraduate education, and research. The Content Validity Ratio (CVR) (Lawshe et al., 1975) was used to assess the instrument’s content validity using expert opinion [11]. The same panel evaluated each questionnaire item by rating it on a 4-point scale (4 = Highly relevant, 3 = Quite relevant but needs rewording, 2 = Somewhat relevant and 1 = Not relevant). The CVR for each item of the scale was calculated using the following formula:

CVR = (ne-N/2)/N/2 (ne = number of experts who agreed; N = total number of experts)

As per criteria given by Lawshe [11], the minimum CVR value for an item to be retained was established and their mean was used to derive the Content Validity Index (CVI). The sentiment analysis of the expert opinion on the TEAL scale was carried out using the qualitative data analysis software (Nvivo 14). The experts’ panel pointed out the risk of ‘ya-saying bias from including only positive statements as per the original TEAL questionnaire. Therefore, 4 out of 20 questions were worded negatively to remove ‘ya’-saying bias.

The adapted TEAL questionnaire (Appendix A) consisted of socio-demographic questions, and self-reporting questions with 20 items categorised under 4 domains: interactive engagement, problem-solving skills, interest, and feedback. Each question had responses on a seven-point Likert scale regarding their perception of the learning activity. The scale was graded as follows (1 = strongly agree, 2 = moderately agree, 3 = slightly agree, 4 = neither agree nor disagree, 5 = slightly disagree, 6 = moderately disagree and 7 = strongly disagree) as per the original TEAL scale. Four open-ended questions were also included for qualitative analysis.

The sample size for the psychometric validation of the adapted TEAL questionnaire was assessed by the Kaiser–Meyer–Olkin (KMO) test and taken as 100 at the 95% confidence interval [12,13]. Considering the 10% non-respondent rate, the final sample size was 110. The questionnaire was administered to the available sample of 113 third-year undergraduate students.

Data were analysed with SPSS (IBM Version 29). The reliability of the adapted TEAL scale was assessed using Cronbach’s Alpha statistic, and the internal consistency of the four domains of the scale was assessed in terms of the overall correlation of each item within the scale [14]. Furthermore, the inter-item and corrected total-item correlation coefficients were used to verify the reliability.

### 2.3. Stage II: Dental Students’ Perception of Technology-Enabled Active Learning

Stage II of the study was conducted with the third-year students of the following year (*n* = 140). The students engaged in the learning task as described previously. The adapted and validated TEAL questionnaire (Appendix A) was administered to the students on completion of the learning task, and the response rate was 84.2.%. Data were analysed using IBM SPSS version 29.0. A non-parametric test (one sample Chi-square test) and an inferential statistical test were used for quantitative data analysis. Nvivo 14 was used for qualitative data analysis. Labels were assigned to research questions such as “positive aspects of contemporary technology-enabled fixed restoration” and “negative aspects of contemporary technology-enabled fixed restoration”. A description-focused coding strategy was utilised based on the available data. Codes were identified under each research question, and four themes were identified based on the clusters generated.

## 3. Results

### 3.1. Stage I: Adaptation and Validation of Questionnaire

The study sample for psychometric property analysis consisted of 102 individuals, with a 97.7% response rate, of which 52.1% were females, 53.2% were 18 to 25 years old, and 40.4% were between 25 and 35 years.

**Figure 1:**
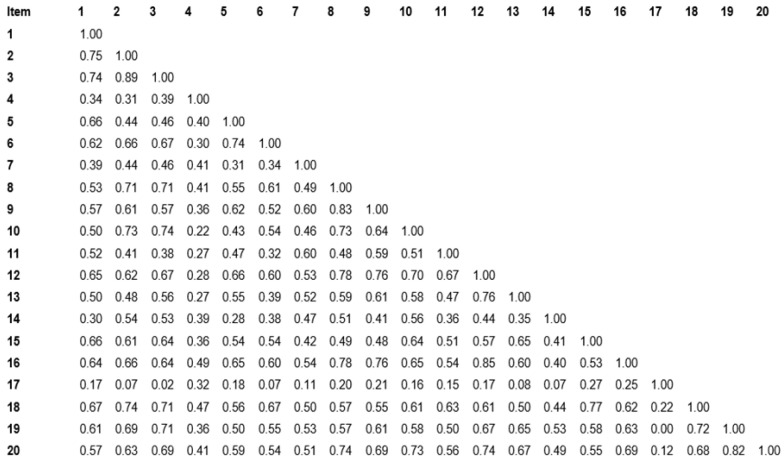
Inter-item correlation matrix.

The sample adequacy for factor analysis resulted in a value of 0.834, and Bartlett’s test of sphericity resulted in a highly significant *p*-value (*p* < 0.001). The inter-item correlation matrix of the adapted TEAL questionnaire (Figure 1) shows an excellent positive correlation around 0.3, 0.4, and 0.5. However, there were very few values higher than 0.7. According to the results of the reliability analysis, the correlation matrix was positive for further analysis as no negative nor zero correlations denote redundancy [14]. Most values were between 0.3 and 0.6, demonstrating a good correlation among the latent variables. Item-total statistics were analysed to obtain the corrected total-item correlation of the adapted TEAL scale. The results are illustrated in Table 1. Almost all the corrected item correlation values were around 0.7 and above the cut-off value of 0.2. This shows that the homogeneity of the adapted TEAL scale is related to our study sample. The Cronbach’s Alpha value for the total scale was 0.952. All the values of items deleted were below the value of 0.96; the results are shown in Table 2. Nunnly (1994) recommended a reliability estimate of 0.7 [13,14]. The TEAL questionnaire was assessed using Cronbach’s Alpha reliability coefficient. The values in each of the four factors ranged from 0.738 to 0.876 (Table 2).

According to expert opinion, question one (which allowed me to respond expediently to my actions, resulting in a fully responsive interaction) did not meet the minimum CVR of 0.62 (11) as it was only 0.43. However, the CVI was 0.74.

The text-based sentiment analysis using the qualitative data obtained from the ten experts on the content of the original TEAL Scale revealed that six experts had positive sentiments, two were very positive, and four were moderately positive. Four experts had negative sentiments, three were moderately negative, and one was very negative in attitude. The factor loadings in Exploratory Factor Analysis (EFA) and structural equation modelling with Confirmatory Factor Analysis (CFA) were assessed to verify the scale’s construct validity. The factor loading statistics are shown in Table 3.

**Figure 2:**
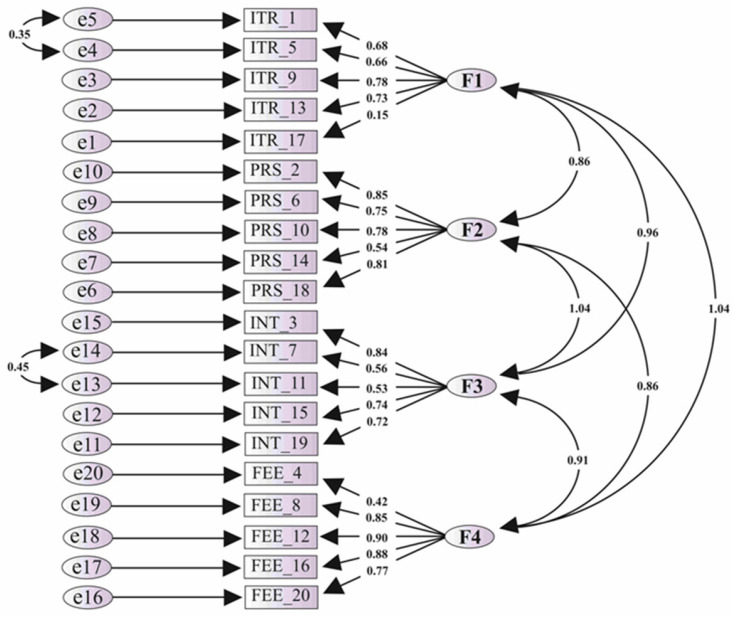
Structural equation model with Confirmatory Factor Analysis.

The CFA was completed with IBM SPSS AMOS Graphic version 27.0, and the Path model is shown in Figure 2. By CFA, the convergent and discriminant validities were also assessed. The original TEAL scale measures the four latent variables of interactive engagement, problem-solving skills, interest, and the same feedback construct of ’technology-enabled active learning’. Thus, higher correlations among different latent variables/factors can be considered excellent discriminant validity [15]. Moreover, it was necessary to note that the pattern of factor loadings was similar to that of the original TEAL scale, verifying its construct validity in our setting. The model fit indices were derived and compared with the desired values. The Relative and Parsimony fit indices are shown in Table 4 and Table 5, respectively.

### 3.2. Stage II: Assessment of Dental Students’ Perception of Technology-Enabled Active Learning

The adapted TEAL questionnaire was carried out on 140 students with a response rate of 84.28%. Most students (93.75%) were positive toward Technology-Enabled Active Learning experiences. Table 6 shows the various degrees of perceived satisfaction. It was evident that there was a statistically significant difference between levels of satisfaction related to all the aspects of the construct.

Qualitative results show that the overall attitudes towards CAD/CAM technology were expressed as positive, citing benefits such as increased efficiency, accuracy, and patient satisfaction. Some students highlighted the importance of integrating modern technology into practice to stay up-to-date and improve clinical outcomes. On ’perceived benefits of CAD/CAM technology’, students described (a) efficiency, noting that CAD/CAM technology allowed for faster procedure; (b) accuracy, appreciating the precise design and fitting capabilities; and (c) patient satisfaction, denoting minimal complaints. Under the ’challenges and concerns’ theme, several respondents mentioned difficulties in learning to use the CAD/CAM software (Dental system 2022) and software glitches, indicating a steep learning curve. Moreover, the expense of equipment and cost of maintenance were identified as other concerns. Under the theme of ’educational value and training needs’, the students acknowledged the need for early exposure to digital technology to prepare them for clinical practice and suggested more hands-on training and instructional videos on digital technology.

## 4. Discussion

Digital workflows have made significant advancements in every discipline in current clinical dentistry, particularly prosthodontics. Implementation of these technologies in dental undergraduate curricula is still considered a novel approach to teach prosthodontics. This is the first Australian study related to UG students’ experience of hands-on digital manufacturing of dental crowns integrated into the preclinical undergraduate curriculum. This innovative approach is expected to enhance their capabilities in using standard CAD/CAM software (Dental system 2022) and tools and build confidence in their abilities with respect to digital technology. In order to assess students’ perceptions of learning, a suitable tool was researched and adapted for the purpose. Observable variables can derive the construct of perception by capturing common or shared variables among several tangible items of a construct [7]. The TEAL scale (Shroff et al. 2019) was selected and adapted to assess the broad construct of students’ perception of similar, but nondental, constructs [10]. The psychometric properties of the adapted TEAL questionnaire were assessed in the Australian university context.

The scale’s EFA and CFA were utilised to assess the construct validity [13,16]. In EFA, Scree plot demonstrated the four-factor structure. The factor loadings with an Eigenvalue of more than one showed 72.9% variability [17], explained by the four factors, further supporting the validity [18]. Moreover, in Principal Component Analysis with Promax rotational method, all the factor loadings exceeded the cut-off value of 0.7. The factor loadings in the original TEAL scale development and validation study were above 0.7. The construct validity was established with structural equation modelling using CFA [19]. In the original study, the fit indices were also assessed; the Relative and Parsimony model fit indices were assessed in the current study. In the Path analysis model, the factor loading for item 17 was 0.16, and for item 4 was 0.46. The model fit indices were improved when the two items were removed from the model.

The reliability of the adapted TEAL scale was ensured in terms of Cronbach’s Alpha value calculated separately for all four factors. In the original scale, the internal consistency ranged from 0.83 to 0.88. In the current study, Cronbach’s Alpha values also exceeded the standard criteria of 0.7, which is the cut-off for minimum reliability [14]. If the item is deleted, Cronbach’s Alpha is below the value for the total scale for 17 items, and this shows that by omitting any of these items, the scale statistics will not improve. However, for item four, the Cronbach’s Alpha value if the item was deleted was the same, and it was slightly higher for item 17. This closely resembled the issue of low factor loadings related to the same items in the CFA Path model. In this scenario, deleting items 4 and 17 would improve the scale. However, according to the literature, retaining all the questions in the scale at the validation study is always advisable since the sample is smaller than the field survey [20]. Therefore, it was decided to keep the items for the current study.

The homogeneity was verified as the corrected total-item correlation values were more than 0.5 except in items 4 (0.48) and 17 (0.195~0.2). The very high inter-item correlation denotes multicollinearity [13]. Moreover, the correlation factors for item 17, where almost all the items denoted redundancy value, indicated that the item “facilitated the exchange of information by engaging with content presented in diverse formats”. This is not well correlated with the others in the ‘Interaction’ construct. According to the results of the reliability analysis, the correlation matrix was positive for further analysis as no negative nor zero correlations denote redundancy [14]. Most values were between the 0.3 to 0.6 range, demonstrating a good correlation among the latent variables. However, the cut-off value was 0.2, and we can be safely assured of the homogeneity of the scale [14]. The respondent rate of the scale was 92.7, as only eight participants did not respond, showing the acceptability of the scale in the current context. The second stage of the study was executed to assess the acceptability of the scale by a sample of end-users, which served as a strength of the study. This was further verified by the supervisors during the activity, who observed that the students were actively and enthusiastically engaged in scanning and CAD/CAM laboratory activities. Their positive feedback in all the domains established the positive perception of Technology-Enabled Active Learning experience.

The second stage of the study revealed that there was no statistically significant difference related to any domain of the adapted TEAL questionnaire across the age or gender of the students. As 60.3% of the cohort are less than 25 years old, 34.5% are between 35 and 45 years old, and only 5.2% are more than 35 years old, the vast majority represent the young generation. This reflects that despite their age and gender, all the students have a positive perception of TEAL. Furthermore, their level of perception showed no significant difference across other demographic information, such as whether they were international or Australian students or had completed any degree before the current dental training.

The qualitative structured analysis provided a comprehensive overview of the qualitative findings regarding the student’s attitudes, perceptions, and experiences with CAD/CAM technology in dentistry. They expressed positive attitudes about the increasing use of digital technology in dental practice, foreseeing its continued growth and integration into clinical workflow. Moreover, the importance of skill development has been emphasised by many students, who recognise the importance of acquiring CAD/CAM skills for future career prospects, leading to delivering high-quality dental care.

Most current undergraduate dental students are from the digital native generation, and therefore have been exposed to different digital technologies throughout their lives [21]. This enables the students to grasp and embrace the skills required to adopt dental digital workflow processes effectively. Developing skills in traditional manual techniques has been a mandatory component of dental education [5]. Therefore, despite the results of this study, which show very positive levels of students’ perceptions, implementing digital workflow in the dental curriculum must occur in conjunction with existing manual training to ensure the best student learning outcomes.

In this study, the students have acknowledged the need for early exposure to digital technology to prepare them for clinical practice and suggested more hands-on training and instructional videos on digital technology. These suggestions were addressed by including more online learning materials in higher year levels. Furthermore, as an advancement of integrating digital workflow in undergraduate training, the students in the fourth and fifth years of dental training are provided with the necessary training in intraoral scanning. Moreover, they are given real-world experience through clinical case application during their undergraduate prosthodontic training.

The design and the time frame did not allow for a pilot study, which could be considered as a limitation of the study. Furthermore, this study was conducted in a well-equipped faculty with well-trained staff. This might affect the generalizability of the method in every part of the world.

Novel digital dental technologies and materials are being introduced into the market at a rapid pace. Developing the capacity to embrace all the new advancements and integrating into undergraduate learning is a significant challenge dental schools are facing today. However, learning basic skills during UG training would awaken the enthusiasm and motivation of future dentists to embrace the digital transformation comfortably. Dental schools/faculties need to update their curricula to ensure essential standards of contemporary learning for students. Furthermore, studies show that dental students believe that the content, structure, and training standards they receive must be improved for developing their competencies [22]. According to the conceptual framework developed by Thomas et al., “for a successful curriculum, curriculum development never really ends” [23]. The student learning task in this study was planned according to the following six steps: problem identification, targeted need assessment, documentation of targeted goals and objectives, finalising educational strategies, implementation, evaluation, and feedback [23]. This study did not assess the potential benefits of integrating digital workflow on students’ learning outcomes. Further research is required to develop and validate specific instruments in the domains of active learning, and potential impacts on students’ learning outcomes. Although the integration of CAD/CAM dentistry into the undergraduate curriculum is a necessity, its incorporation should be supported by robust evidence. The existing UG curricula are already rigorous, skill-intensive, and deadline-driven. However, as the results of this study suggest, curricular revisions with respect to digital technology are warranted, including the digital workflow within the UG prosthodontics training programme.

## 5. Conclusions

The adapted TEAL questionnaire can be recommended for assessing students’ perceptions on implementation of digital workflow in the UG dental curriculum in a similar study setting. Most students positively perceived Technology-Enabled Active Learning. Overall, respondents viewed digital technology positively, acknowledging its potential to improve efficiency, accuracy, and patient satisfaction in dental practice. Curricular revisions to include digital technology are warranted, including the digital workflow within the UG prosthodontics training programme. The authors recommend that future research assess the influence of exposure to a structured training in clinicians’ ability to embrace digital workflow in their practice.

## Figures and Tables

**Table 1:** Item-total statistics: Corrected total-item correlation and Cronbach’s Alpha if the item is deleted.

Item	Corrected Total-Item Correlation	Cronbach’s Alpha if the Item is Deleted
1_Respond expediently for interaction	0.737	0.948
2_Generate ideas	0.786	0.947
3_Engage in dialogue	0.801	0.947
4_Timely feedback	0.481	0.952
5_Skilful interaction	0.679	0.949
6_Systematic problem-solving	0.681	0.949
7_Exploring different issues	0.613	0.951
8_Track of my performance	0.815	0.947
9_Active engagement user- interface	0.792	0.947
10_Encouraged critical thinking	0.768	0.948
11_Piqued my curiosity	0.636	0.950
12_Prompt feedback on progression	0.841	0.946
13_Interaction encouraged	0.697	0.948
14_Analysing views in broader contexts	0.561	0.951
15_Held my attention	0.725	0.948
16_Tracking my progression	0.832	0.946
17_Facilitated exchange information	0.195	0.957
18_Define the problem sys.	0.806	0.948
19_Encouraged to exert effort	0.772	0.947
20_Responses for understanding	0.822	0.947

**Table 2:** Cronbach’s Alpha reliability coefficient for latent factors.

Construct	No. of Items	Cronbach’s Alpha
Interactive engagement (ITR)	5	0.738
Problem-solving skills (PRSs)	5	0.869
Interest (INT)	5	0.844
Feedback (FEE)	5	0.876
Total Cronbach’s Alpha Value	0.952	

**Table 3:** Factor loading statistics of the adapted TEAL scale.

Item	Latent Variable	Factor Loading
Interactive engagement (ITR)		
1	Allowed me to respond expediently to my actions, resulting in a fully responsive interaction	0.76
5	Enabled me to skilfully interact with the features in a responsive manner	0.70
9	Allowed me to actively engage with the user interface in a way that promotes dialogue	0.75
13	Helped me to interact more effectively with peers through an engaging interface	0.63
17	Has not facilitated the exchange of information by engaging with content presented in diverse formats	0.73
Problem-solving skills (PRSs)		
2	Allowed me to generate ideas methodically by contributing information from multiple viewpoints	0.87
6	Enabled me to solve a problem systematically by taking into account different points of view	0.74
10	Encouraged me to think critically about the broader concepts related to the problem	0.67
14	Prevented me analysing my own views and their wider contexts in order to draw firm conclusions	0.73
18	Allowed me to define the problem systematically by viewing it from different angles in an effort to find possible solutions	0.77
Interest (INT)		
3	Allowed me to engage in thought-provoking dialogue with points of view that challenged my perspectives	0.88
7	Prevented me exploring a variety of different issues that I may not have otherwise considered	0.64
11	Piqued my curiosity by exploring various options when navigating the user interface	0.63
15	Held my attention by challenging me to look into issues that I may not have otherwise thought of	0.64
19	Encouraged me to exert effort in the face of difficulty by persisting at tasks I found challenging	0.68
Feedback (FEE)		
4	Prevented me receiving timely feedback that would have helped me improve my performance	0.75
8	Enabled me to receive inputs so that I was able to keep track of my performance	0.71
12	Allowed me to receive prompt feedback so that I was aware of my progression towards knowledge acquisition	0.86
16	Allowed me to receive prompt feedback, so that I was aware of my own progression towards mastery of my skill development	0.74
20	Enabled me to receive responses that allowed further understanding	0.71

**Table 4:** Absolute model fit indices of the adapted TEAL scale obtained by Confirmatory Factor Analysis.

X^2^	df	*p*	RMSEA	GFI	AGFI	SRMR
66.33	64	0.033	0.055	0.837	0.836	0.070

**Table 5:** Relative and Parsimony model fit indices of the adapted TEAL scale obtained by Confirmatory Factor Analysis.

Relative Fit Indices		Parsimony Fit Indices	
CFI	NFI	PGFI	PNFI
0.979	0.861	0.498	0.570

**Table 6:** Student’s perceptions on Technology-Enabled Active Learning (N= 116).

Construct	Ques. No	Percentage of Students with the Perception							X^2^	*p*
		1	2	3	4	5	6	7		
Interactive engagement (ITR)	1	19.8	40.5	24.1	7.8	3.4	2.6	1.7	103.172	<0.001
	5	26.7	50.0	18.1	3.4	1.7	0	0	90.293	<0.001
	9	24.1	48.3	25.0	2.6	0	0	0	48.483	<0.001
	13	26.7	31.9	28.4	12.1	0.9	0	0	39.862	<0.001
	17	8.6	13.8	11.2	12.9	12.1	25.9	15.5	14.948	0.021
ITR Composite score	Mean = 13.33 SD = 3.464									
Problem-solving skills (PRSs)	2	34.5	36.2	23.3	6.0	0	0	0	26.828	<0.001
	6	35.3	29.3	27.6	7.8	0	0	0	19.931	<0.001
	10	38.8	44.8	12.9	3.4	0	0	0	55.379	<0.001
	14	3.4	6.9	10.3	13.8	17.2	33.6	14.7	46.328	<0.001
	18	25.0	45.7	29.3	0	0	0	0	8.293	0.016
PRS Composite score	Mean = 12.88 SD = 2.810									
Interest (INT)	3	37.9	38.8	15.5	7.8	0	0	0	34.552	<0.001
	7	1.7	12.1	11.2	11.2	9.5	29.3	25.0	44.276	<0.001
	11	58.6	31.0	9.5	9.0	0	0	0	92.345	<0.001
	15	40.5	40.5	13.8	5.2	0	0	0	46.414	<0.001
	19	31.9	35.3	26.7	6.0	0	0	0	24.000	<0.001
INT Composite score	Mean = 12.39 SD = 2.695									
Feedback (FEE)	4	0.9	12.9	17.2	12.9	13.8	25.0	17.2	25.690	<0.001
	8	21.6	47.4	24.1	6.9	0	0	0	39.103	<0.001
	12	32.8	39.7	23.3	3.4	0.9	0	0	69.603	<0.001
	16	26.7	43.1	19.8	6.9	3.4	0	0	59.431	<0.001
	20	33.6	33.6	31.9	0.9	0	0	0	36.138	<0.001
Fee Composite score	Mean = 13.03 SD = 2.928									
TEAL Composite score	Mean = 43.06 SD = 7.859									

## Data Availability

The raw data supporting the conclusions of this article will be made available by the authors on request.

## Abbreviations

The following abbreviations are used in this manuscript:

AGFI	Adjusted Goodness of Fit Index
CAD	Computer-aided design
CAM	Computer-aided manufacturing
CFA	Confirmatory Factor Analysis
CFI	Comparative Fit Index
CI	Confidence interval
CVI	Content Validity Index
CVR	Content Validity Ratio
EFA	Exploratory Factor Analysis
GFI	Goodness of Fit Index
NFI	Non-Normal Fit Index
PGFI	Parsimony Goodness of Fit Index
PNFI	Parsimony Normed Fit Index
PMMA	Poly methyl methacrylate
RMSEA	Root Mean Square Estimate of Approximation
SRMR	Standardised Root Mean Square Residual
TEAL	Technology-Enabled Active Learning
UG	Undergraduate

## Appendix A

The Questionnaire: Implementation of a digital workflow in prosthodontic education curriculum

Section A: About YOU

Please **circle** your response

Are you?

1. Male
2. Female
3. Non-binary
4. Prefer not to say

What is your age?

1. 18–25 years
2. 26–35 years
3. 36–45 years
4. 46–55 years
5. Above 55 years

Where did you attend High School?

1. Australian
2. International
3. Prefer not to say

Have you studied at a university before commencing the Bachelor of Dental Health Science?

1. Yes
2. No
3. Unsure
4. Prefer not to say

Section B: Your experience of creating a Gold Crown using CAD/CAM approach

Please indicate the most appropriate response (1–7 as per below) to the following statements regarding “Using Computer Aided Design and Computer Aided Manufacturing” in the course 3060DOH.

1. Strongly agree
2. Moderately agree
3. Slightly agree
4. Neither agree nor disagree
5. Slightly disagree
6. Moderately disagree
7. Strongly disagree

**Table:**

	**Statement**	**1**	**2**	**3**	**4**	**5**	**6**	**7**
1.	Allowed me to respond expediently to my actions, resulting in a fully responsive interaction							
2.	Allowed me to methodically generate ideas by contributing information from multiple viewpoints							
3.	Allowed me to engage in thought-provoking dialogue with points of view that challenged my perspectives							
4.	Prevented me receiving timely feedback that would have helped me improve my performance							
5.	Enabled me to skilfully interact with the features in a responsive manner							
6.	Enabled me to solve a problem systematically by taking into account different points of view							
7.	Prevented me exploring a variety of different issues that I may not have otherwise considered							
8.	Enabled me to receive inputs, so that I was able to keep track of my own performance							
9.	Allowed me to actively engage with the user- interface in a way that promotes dialogue							
10.	Encouraged me to think critically about the broader concepts related to the problem							
11.	Piqued my curiosity by exploring various options when navigating the user interface							
12.	Allowed me to receive prompt feedback, so that I was aware of my own progression towards knowledge acquisition							
13.	Helped me to interact more effectively with peers through an engaging interface							
14.	Prevented me analysing my own views and their wider contexts in order to draw firm conclusions							
15.	Held my attention by challenging me to look into issues that I may not have otherwise thought of							
16.	Allowed me to receive prompt feedback, so that I was aware of my own progression towards mastery of my skill development							
17.	Has not facilitated the exchange of information by engaging with content presented in diverse formats							
18.	Allowed me to define the problem systematically by viewing it from different angles in an effort to find possible solutions							
19.	Encouraged me to exert effort in the face of difficulty by persisting at tasks I found challenging							
20.	Enabled me to receive responses that allow further understanding							

**Section C: Your detailed experience**

Q1. After engaging and using the CAD/CAM process as an undergraduate student: Do you think you would be more likely to incorporate this technology into your clinical practice when you graduate?
  (a) Yes  (b) No  (c) Unsure-If **YES**, please elaborate on your answer.
----------------------------------------------------------------------------------------------------------------------------------------------------------------------------------------------------------------------------------------------------------------------------------------------------------------------------------------------------------------------------------------------------------------------------------------------------------------------------------------------------------------------------------------------------------------------------
Q2. Describe three positive aspects resulting from applying and creating a traditional and a contemporary approach to a single tooth fixed restoration.
----------------------------------------------------------------------------------------------------------------------------------------------------------------------------------------------------------------------------------------------------------------------------------------------------------------------------------------------------------------------------------------------------------------------------------------------------------------------------------------------------------------------------------------------------------------------------
Q3. Describe three of the **least positive** aspects resulting from applying and creating a traditional and a contemporary approach to a single tooth fixed restoration.
----------------------------------------------------------------------------------------------------------------------------------------------------------------------------------------------------------------------------------------------------------------------------------------------------------------------------------------------------------------------------------------------------------------------------------------------------------------------------------------------------------------------------------------------------------------------------
